# Supplementary material for: Observational study of agreement between attending and trainee physicians on the surprise question: “Would you be surprised if this patient died in the next 12 months?”
Source: PLoS One. 2021 Feb 25;16(2):e0247571. doi: 10.1371/journal.pone.0247571 (PMC7906409; doi:10.1371/journal.pone.0247571)
Supplement: S1 File — (DOCX) [file pone.0247571.s001.docx]

Preliminary confidential draft. Comments welcome

2020-07-02

**Supplement: Agreement Between Attending and Trainee Physicians on the Surprise Question**

Key words (MeSH): terminal care, palliative care, critical care, prognostication,

general internal medicine

Yarnell, Christopher J

Jewell, L

Astell A,

Pinto, R

Devine, L

Downar, J

Ilan, R

Rawal, S

Wong, N

You, J

Fowler, RA

Contents

[Details about participating hospitals 3](#_Toc45011517)

[Details of Bayesian Modeling 3](#_Toc45011518)

[Prior distributions 3](#_Toc45011519)

[Computational details 4](#_Toc45011520)

[R code 4](#_Toc45011521)

[Primary model for Cohen’s kappa 5](#_Toc45011522)

[Figure E1: Primary model coefficient distributions and trace plots 6](#_Toc45011523)

[Cohen’s kappa from the posterior distributions of coefficients 7](#_Toc45011524)

[Secondary model for 12-month mortality 8](#_Toc45011525)

[Figure E2: Secondary model coefficient distributions and trace plots 9](#_Toc45011526)

[R code for sensitivity, specificity, and likelihood ratios 9](#_Toc45011527)

[Exploratory model for factors associated with agreement 11](#_Toc45011528)

[Additional Tables 13](#_Toc45011529)

[Table E1 – Trainee physician surprise question responses compared with mortality at 12 months 13](#_Toc45011530)

[Table E2 – Attending physician surprise question responses compared with mortality at 12 months 13](#_Toc45011531)

[Table E3 – Test characteristics of surprise question responses for predicting mortality at 12 months 14](#_Toc45011532)

[Table E4 – Surprise question responses with respect to admission versus 12 months 14](#_Toc45011533)

[Table E5 – Hospital admission surprise question responses of attending and resident physicians 15](#_Toc45011534)

[References 16](#_Toc45011535)

# Details about participating hospitals

The seven participating hospitals were Kingston General Hospital in Kingston, Ontario, Canada; Hamilton General Hospital in Hamilton, Ontario, Canada; St Michael’s Hospital, Mount Sinai Hospital, Toronto General Hospital, and Sunnybrook Health Sciences Centre all in Toronto, Ontario, Canada. Each hospital runs an academic general internal medicine inpatient program structured in teams with the following approximate composition: 15-30 patients, one attending physician, one senior (postgraduate year 2 or higher) resident, and multiple junior trainees (first year residents, medical students).

# Details of Bayesian Modeling

The initial protocol and statistical plan used a frequentist calculation of the Cohen’s kappa that assumed the surprise question response for every patient was independent. There are no frequentist methods that allow for regression by covariates or account for clustering in calculation of the Cohen’s kappa, and instead most studies that attempt this instead use agreement or another outcome instead of the Cohen’s kappa.

While the study was navigating Research Ethics Board approval and gathering data at multiple sites, the lead author gained facility with Bayesian analyses and saw how a Bayesian approach would allow for clustering at the level of the physician pair and regression according to patient characteristics while still maintaining the Cohen’s kappa as the primary statistical output.

## Prior distributions

This analysis used minimally informative prior distributions including wide normal distributions for the coefficients in logit-space (mean 0, standard deviation 5) and a half-student-t prior with 3 degrees of freedom and scale parameter 10.^2–4^

## Computational details

The analyses were coded in RStudio v 1.2.5033 using R v 3.6.3^5^ using the tidyverse^6^ and brms^2^ packages.

## R code

All R code used to conduct the analysis is available upon request (email [christopher.yarnell@sinaihealth.ca](mailto:christopher.yarnell@sinaihealth.ca)). Much of the code is focused on data wrangling, but the code for the primary model is available below.

## Primary model for Cohen’s kappa

Here we include code for the primary model.

b2 <- brm(
 data = d,
 family = categorical(link = logit),
 response_12mo ~ 1 + (1 | pair_id),
 prior= c(prior_string("normal(0,5)", class = "Intercept")),
 iter = 4000, warmup = 1000, chains = 4, cores = 4
 )

summary(b2)

Family: categorical

Links: muNY = logit; muYN = logit; muYY = logit

Formula: response_12mo ~ 1 + (1 | pair_id)

Data: d (Number of observations: 546)

Samples: 4 chains, each with iter = 4000; warmup = 1000; thin = 1;

total post-warmup samples = 12000

Group-Level Effects:

~pair_id (Number of levels: 30)

Estimate Est.Error l-95% CI u-95% CI Rhat

sd(muNY_Intercept) 0.83 0.25 0.40 1.36 1.00

sd(muYN_Intercept) 0.95 0.31 0.43 1.64 1.00

sd(muYY_Intercept) 0.47 0.19 0.08 0.87 1.00

Bulk_ESS Tail_ESS

sd(muNY_Intercept) 3770 5978

sd(muYN_Intercept) 3916 5223

sd(muYY_Intercept) 3054 3026

Population-Level Effects:

Estimate Est.Error l-95% CI u-95% CI Rhat

muNY_Intercept -1.27 0.22 -1.74 -0.86 1.00

muYN_Intercept -2.14 0.29 -2.79 -1.63 1.00

muYY_Intercept -0.73 0.15 -1.03 -0.45 1.00

Bulk_ESS Tail_ESS

muNY_Intercept 5591 6718

muYN_Intercept 6535 6082

muYY_Intercept 9123 8161

Samples were drawn using sampling(NUTS). For each parameter, Bulk_ESS

and Tail_ESS are effective sample size measures, and Rhat is the potential

scale reduction factor on split chains (at convergence, Rhat = 1).

The model output shows R-hat values equal to 1 and reasonable effective samples sizes (ESS), consistent with model convergence.

## Figure E1: Primary model coefficient distributions and trace plots


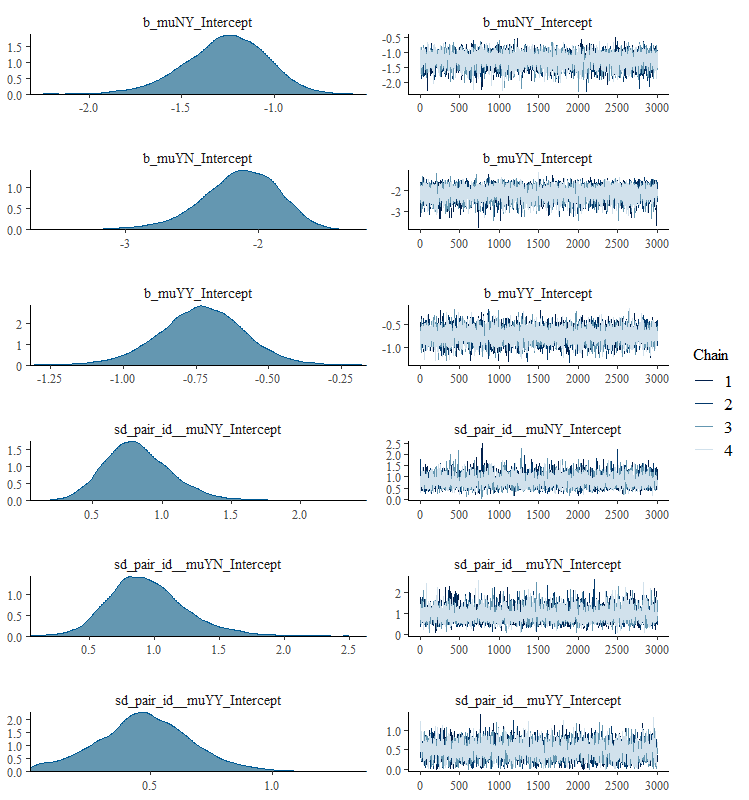


This figure shows the posterior distributions of the regression coefficients (top 3) and variance parameters (bottom 3) corresponding to the surprise question responses (lefthand column) and the trace plots of the Markov-Chain Monte-Carlo simulations (righthand column). The trace plots show stable convergence. A “No” response from both physicians is the reference category.

##

## Cohen’s kappa from the posterior distributions of coefficients

Different from the frequentist approach, the use of Bayesian methods and MCMC simulation gives a full posterior distribution for each coefficient. This can be used to calculate the posterior probability distributions of a patient being in each state (Yes – Yes, Yes – No, No – Yes, No – No) and from those posterior probability distributions we can calculate the posterior probability distribution of the Cohen’s kappa. Note that we do not include variation according to physician pair in this calculation – this is an estimate of the mean Cohen’s kappa and 95% credible interval, not an estimate of the predictive distribution for an individual physician pair’s Cohen’s kappa.

post <- **posterior_samples**(b2)
*# post is now a matrix with the MCMC output for each coefficient*

*# calculate normalizing coefficient*
total_sum <- 1**+** **exp**(post**$**b_muNY_Intercept) **+** **exp**(post**$**b_muYN_Intercept) **+** **exp**(post**$**b_muYY_Intercept)

*# calculate probability of being in each state*
p.a <- 1**/**total_sum *# this is the reference category, NN*
p.b <- **exp**(post**$**b_muNY_Intercept)**/**total_sum
p.c <- **exp**(post**$**b_muYN_Intercept)**/**total_sum
p.d <- **exp**(post**$**b_muYY_Intercept)**/**total_sum

*# next we calculate Cohen's kappa in the usual way,*
*# except we are using the whole posterior distribution*
p.obs <- p.a **+** p.d
p.exp <- (p.a**+**p.b)*****(p.a**+**p.c)**+**(p.c**+**p.d)*****(p.b**+**p.d)
p.kappa <- (p.obs **-** p.exp)**/**(1**-**p.exp)

**quantile**(p.kappa, **c**(0.025, 0.5, 0.975)) *# median and 95% credible interval*

## Secondary model for 12-month mortality

Here we include code for model for 12-month mortality, accounting for clustering.

*# here we use the surprise responses (YY, YN, NY, or NN) as a categorical predictor of mortality outcome*

b4 <- **brm**(
 data = d,
 family = binomial,
 death_12mo **|** **trials**(1) **~** 1 **+** response_12mo **+** ( 1 **|** pair_id ),
 prior= **c**(**set_prior**("normal(0,5)", class = "Intercept"),
 **set_prior**("normal(0,5)", class = "b")),
 iter = 4000, warmup = 1000, chains = 4, cores = 4)

quantiles <- **function**(x){**quantile**(x, **c**(0.025, 0.5, 0.975))}

*# posterior samples of the probability of mortality according to each of the 4 possible surprise question paired responses (YY, NY, YN, or NN)*
post4 <- **posterior_samples**(b4)
p.m.nn <- **inv_logit_scaled**(post4**$**b_Intercept)
p.m.ny <- **inv_logit_scaled**(post4**$**b_Intercept**+**post4**$**b_response_12moNY)
p.m.yn <- **inv_logit_scaled**(post4**$**b_Intercept **+** post4**$**b_response_12moYN)
p.m.yy <- **inv_logit_scaled**(post4**$**b_Intercept**+**post4**$**b_response_12moYY)

*# next we have to calculate the probability of each response in order to separate out the estimates of SMRs and attending physicians*
p.nn <- **mean**(b4**$**data**$**response_12mo **==** "NN")
p.ny <- **mean**(b4**$**data**$**response_12mo **==** "NY")
p.yn <- **mean**(b4**$**data**$**response_12mo **==** "YN")
p.yy <- **mean**(b4**$**data**$**response_12mo **==** "YY")

*# now we calculate the probability of death given a particular SQ response by either the SMR or the attending physician. It is the probability of death AND having a given SQ response by a given physician DIVIDED BY the probability of that SQ response by that physician (Bayes' rule)*
p.smr.y <- (p.m.ny*****p.ny **+** p.m.yy*****p.yy)**/**(p.ny**+**p.yy)
p.ap.y <- (p.m.yn*****p.yn **+** p.m.yy*****p.yy)**/**(p.yn **+** p.yy)
p.smr.n <- (p.m.yn*****p.yn **+** p.m.nn*****p.nn)**/**(p.yn **+** p.nn)
p.ap.n <- (p.m.ny*****p.ny **+** p.m.nn*****p.nn)**/**(p.ny **+** p.nn)

**quantiles**(p.smr.y)
**quantiles**(p.ap.y)
**quantiles**(p.smr.n)
**quantiles**(p.ap.n)

## Figure E2: Secondary model coefficient distributions and trace plots


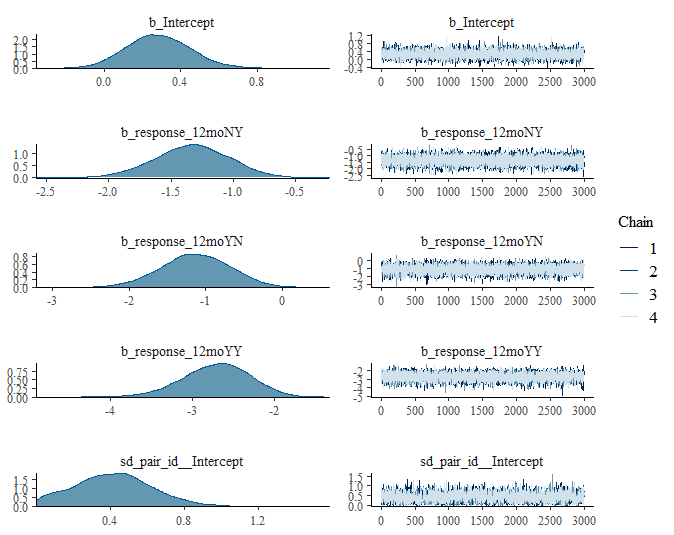


This figure shows the posterior distributions and trace plots of the regression coefficients for each surprise question response in modelling the outcome of mortality (top 4, response “NN” is the intercept or reference) and the variance parameter across physician pairs (bottom). The trace plots show stable convergence.

## R code for sensitivity, specificity, and likelihood ratios

Below we include the R code for calculating the test characteristics for a 12-month surprise question response of “No” and the outcome 12-month mortality by physician type (attending vs trainee).

# to get the adjusted sensitivity and specificity we have to use the predicted mortality from our model. Each prediction is a draw from the posterior
distribution of coefficients derived by our model so we generate 1000
predicted outcomes for each patient.
# setting the re_formula term to NA gives us the performance of the average
pair after accounting for clustering
predicted_responses <- predict(b4, summary = F,nsamples = 1000, re_formula = NA)

d_mort <- b4$data

# SMR "N" responses (second character)
SMR <- (d_mort$response_12mo == "YN") | (d_mort$response_12mo == "NN")
# AP "N" responses (first character)
AP <- (d_mort$response_12mo == "NY") | (d_mort$response_12mo == "NN")

# this function calculates the test characteristics for SQ response "NO" and the outcome 12-month Mortality compared to a single set of predicted outcomes
Bayes2by2 <- function(x, Y){

 sens <- sum(x[Y == T])/sum(x)

 spec <- sum(x[Y == F] == 0)/sum(x == 0)

 LRpos <- sens/(1-spec)
 LRneg <- (1-sens)/spec

 c("sens" = sens,
 "spec" = spec,
 "LRpos" = LRpos,
 "LRneg" = LRneg)
}

# Here we use the apply() function to generate an estimate of sens / spec / LR+ / LR- for each of the 1000 sets of outcomes we predicted from the posterior distribution
SMR_2b2 <- apply(predicted_responses, 1, Bayes2by2, SMR)
AP_2b2 <- apply(predicted_responses, 1, Bayes2by2, AP)

round(data.frame(
 SMR_sens = quantiles(SMR_2b2[1,]),
 AP_sens = quantiles(AP_2b2[1,]),
 SMR_spec = quantiles(SMR_2b2[2,]),
 AP_spec = quantiles(AP_2b2[2,]),
 SMR_LRp = quantiles(SMR_2b2[3,]),
 AP_LRp = quantiles(AP_2b2[3,]),
 SMR_LRn = quantiles(SMR_2b2[4,]),
 AP_LRn = quantiles(AP_2b2[4,])),2)

## Exploratory model for factors associated with agreement

Here we include code and output for model for 12-month mortality, accounting for clustering. The age and creatinine are centered at their mean and scaled by their standard deviation (code not shown).

b25 <- **brm**(
 data = d,
 family = **categorical**(link = logit),
 response_12mo **~** 1 **+** ( 1 **|** pair_id) **+**
 gender **+**
 **s**(age, k = 4, bs = "cr") **+**
 functional **+** GofC_CPR **+**
 PMHX_CV **+** PMHX_Resp **+** PMHX_CKD **+** PMHX_Cancer **+**
 Dxx_CV **+** Dxx_Infxn **+** Dxx_AKI_lytes **+**
 Dxx_Neuro,
 prior = **c**(**set_prior**("normal(0,10)", class = "Intercept"),
 **set_prior**("normal(0,10)",class = "b"),
 **prior_string**("cauchy(0,1)", class = "sd",
 dpar = "muNY"),
 **prior_string**("cauchy(0,1)", class = "sd",
 dpar = "muYN"),
 **prior_string**("cauchy(0,1)", class = "sd",
 dpar = "muYY")),
 iter = 7500, warmup = 5000, chains = 4, cores = 4,
 control = **list**(adapt_delta = 0.95)
)
**print**(b25)

## Family: categorical
## Links: muNY = logit; muYN = logit; muYY = logit
## Formula: response_12mo ~ 1 + (1 | pair_id) + gender + s(age, k = 4, bs = "cr") + functional + GofC_CPR + PMHX_CV + PMHX_Resp + PMHX_CKD + PMHX_Cancer + Dxx_CV + Dxx_Infxn + Dxx_AKI_lytes + Dxx_Neuro
## Data: d (Number of observations: 544)
## Samples: 4 chains, each with iter = 7500; warmup = 5000; thin = 1;
## total post-warmup samples = 10000
##
## Smooth Terms:
## Estimate Est.Error l-95% CI u-95% CI Rhat Bulk_ESS Tail_ESS
## sds(muNY_sage_1) 2.89 3.20 0.35 11.63 1.00 2859 3865
## sds(muYN_sage_1) 1.51 2.04 0.04 7.40 1.00 3512 5101
## sds(muYY_sage_1) 1.06 1.78 0.02 6.02 1.00 3116 3897
##
## Group-Level Effects:
## ~pair_id (Number of levels: 30)
## Estimate Est.Error l-95% CI u-95% CI Rhat Bulk_ESS Tail_ESS
## sd(muNY_Intercept) 0.92 0.25 0.49 1.47 1.00 3464 5822
## sd(muYN_Intercept) 0.90 0.34 0.23 1.61 1.00 2581 2238
## sd(muYY_Intercept) 0.69 0.24 0.19 1.18 1.00 2554 2542
##
## Population-Level Effects:
## Estimate Est.Error l-95% CI u-95% CI Rhat Bulk_ESS
## muNY_Intercept -1.32 0.63 -2.56 -0.10 1.00 8708
## muYN_Intercept -2.69 0.91 -4.56 -0.95 1.00 8602
## muYY_Intercept -3.40 0.75 -4.92 -1.98 1.00 8259
## muNY_genderM -0.18 0.28 -0.73 0.36 1.00 11981
## muNY_functionalD 0.06 0.43 -0.79 0.93 1.00 8131
## muNY_functionalI 0.25 0.44 -0.61 1.13 1.00 7675
## muNY_functionalU -0.08 0.48 -1.00 0.89 1.00 7980
## muNY_GofC_CPRY 0.63 0.32 0.01 1.24 1.00 12589
## muNY_PMHX_CVTRUE -0.09 0.38 -0.84 0.65 1.00 12077
## muNY_PMHX_RespTRUE 0.14 0.35 -0.55 0.80 1.00 9840
## muNY_PMHX_CKDTRUE -0.45 0.43 -1.32 0.35 1.00 11980
## muNY_PMHX_CancerTRUE -0.53 0.33 -1.19 0.11 1.00 13361
## muNY_Dxx_CVTRUE 0.00 0.39 -0.78 0.75 1.00 13309
## muNY_Dxx_InfxnTRUE -0.16 0.31 -0.77 0.44 1.00 12167
## muNY_Dxx_AKI_lytesTRUE -1.00 0.50 -2.01 -0.05 1.00 11721
## muNY_Dxx_NeuroTRUE -0.17 0.38 -0.94 0.57 1.00 12368
## muYN_genderM 0.03 0.39 -0.72 0.80 1.00 11321
## muYN_functionalD -0.53 0.77 -2.02 1.03 1.00 7779
## muYN_functionalI 0.84 0.69 -0.45 2.29 1.00 7393
## muYN_functionalU 0.87 0.69 -0.44 2.28 1.00 7621
## muYN_GofC_CPRY 0.37 0.47 -0.53 1.32 1.00 9637
## muYN_PMHX_CVTRUE 0.08 0.52 -0.94 1.11 1.00 9035
## muYN_PMHX_RespTRUE 0.25 0.50 -0.75 1.23 1.00 11150
## muYN_PMHX_CKDTRUE -1.26 0.88 -3.16 0.23 1.00 10869
## muYN_PMHX_CancerTRUE -0.98 0.53 -2.08 0.01 1.00 12168
## muYN_Dxx_CVTRUE -0.78 0.72 -2.33 0.49 1.00 12674
## muYN_Dxx_InfxnTRUE 0.24 0.41 -0.58 1.04 1.00 12389
## muYN_Dxx_AKI_lytesTRUE -0.18 0.60 -1.40 0.94 1.00 11079
## muYN_Dxx_NeuroTRUE -0.49 0.65 -1.86 0.72 1.00 13555
## muYY_genderM -0.14 0.28 -0.67 0.41 1.00 12342
## muYY_functionalD -0.02 0.55 -1.07 1.09 1.00 8150
## muYY_functionalI 1.42 0.50 0.46 2.43 1.00 7327
## muYY_functionalU 0.34 0.53 -0.71 1.41 1.00 7615
## muYY_GofC_CPRY 2.18 0.47 1.31 3.17 1.00 12582
## muYY_PMHX_CVTRUE 0.53 0.38 -0.21 1.28 1.00 11160
## muYY_PMHX_RespTRUE -0.45 0.43 -1.31 0.39 1.00 11720
## muYY_PMHX_CKDTRUE -0.49 0.43 -1.37 0.36 1.00 12755
## muYY_PMHX_CancerTRUE -1.16 0.39 -1.95 -0.42 1.00 12211
## muYY_Dxx_CVTRUE 0.36 0.43 -0.49 1.20 1.00 13080
## muYY_Dxx_InfxnTRUE 0.07 0.31 -0.54 0.68 1.00 11960
## muYY_Dxx_AKI_lytesTRUE 0.34 0.40 -0.45 1.12 1.00 11548
## muYY_Dxx_NeuroTRUE 0.01 0.46 -0.90 0.89 1.00 10727
## muNY_sage_1 -0.87 0.53 -1.90 0.19 1.00 6573
## muYN_sage_1 -0.36 0.64 -1.60 0.91 1.00 7670
## muYY_sage_1 -1.84 0.44 -2.75 -1.02 1.00 7046
##
## Samples were drawn using sampling(NUTS). For each parameter, Bulk_ESS
## and Tail_ESS are effective sample size measures, and Rhat is the potential
## scale reduction factor on split chains (at convergence, Rhat = 1).

# Additional Tables

Here we include additional tables comparing attending and trainee physician responses and test characteristics for 12 month mortality and a table of hospital mortality surprise question responses.

## Table E1 – Trainee physician surprise question responses compared with mortality at 12 months

|  | Trainee Physician | | | |
| --- | --- | --- | --- | --- |
| Patient mortality at 12 months |  | “No, I would not be surprised if this patient died in the next 12 months.” | “Yes, I would be surprised if this patient died in the next 12 months.” |  |
|  | Dead | 132 (32%) | 28 (7%) | 160 (38%) |
|  | Alive | 118 (28%) | 139 (33%) | 257 (62%) |
|  |  | 250 (60%) | 167 (40%) | 417* |

## Table E2 – Attending physician surprise question responses compared with mortality at 12 months

|  | Attending Physician | | | |
| --- | --- | --- | --- | --- |
| Patient mortality at 12 months |  | “No, I would not be surprised if this patient died in the next 12 months.” | “Yes, I would be surprised if this patient died in the next 12 months.” |  |
|  | Dead | 143 (34%) | 17 (4%) | 160 (38%) |
|  | Alive | 155 (37%) | 102 (25%) | 257 (62%) |
|  |  | 298 (71%) | 119 (29%) | 417 |

## Table E3 – Test characteristics of surprise question responses for predicting mortality at 12 months

| Characteristic | Median (95% credible interval) | |
| --- | --- | --- |
|  | Trainee physicians | Attending physicians |
| Sensitivity | 0.83 (0.76 to 0.90) | 0.90 (0.83 to 0.96) |
| Specificity | 0.55 (0.49 to 0.61) | 0.40 (0.36 to 0.45) |
| Positive likelihood ratio | 1.84 (1.55 to 2.22) | 1.51 (1.30 to 1.72) |
| Negative likelihood ratio | 0.31 (0.17 to 0.48) | 0.25 (0.10 to 0.46) |

## Table E4 – Surprise question responses with respect to admission versus 12 months

| “Would you be surprised if this patient died…” | | “…in the next 12 months?” | | | |
| --- | --- | --- | --- | --- | --- |
|  |  | NN | NY | YN | YY |
| “…on this hospital admission?” | NN | 57 (10%) | 0 (0%) | 1 (0%) | 0 (0%) |
|  | NY | 43 (8%) | 23 (4%) | 0 (0%) | 1 (0% |
|  | YN | 33 (6%) | 0 (0%) | 5 (1%) | 2 (0%) |
|  | YY | 139 (25%) | 73 (13%) | 38 (7%) | 131 (24%) |

This table shows the contrasts between responses to the surprise question for hospital admission versus next 12 months. N = No, Y = Yes, and attending physician responses are the first initial. For example, responses YN means that the attending physician responded “Yes” and the trainee physician responded “No.”

## Table E5 – Hospital admission surprise question responses of attending and resident physicians

|  | Attending Physician | | | |
| --- | --- | --- | --- | --- |
| Trainee Physician |  | “No, I would not be surprised if this patient died on this hospital admission.” | “Yes, I would be surprised if this patient died on this hospital admission.” |  |
|  | “No, I would not be surprised if this patient died on this hospital admission.” | 58 (11%) | 67 (12%) | 125 (23%) |
|  | “Yes, I would be surprised if this patient died on this hospital admission.” | 40 (7%) | 381 (70%) | 421 (77%) |
|  |  | 98 (18%) | 448 (82%) | 546 (100%) |

# References

1. Quinn KL, Stall NM, Yao Z, et al. The risk of death within 5 years of first hospital admission in older adults. *CMAJ*. 2019;191(50):E1369-E1377. doi:10.1503/cmaj.190770

2. Bürkner PC. brms: An R package for Bayesian multilevel models using Stan. *J Stat Softw*. 2017;80(1):1-28. doi:10.18637/jss.v080.i01

3. McElreath R. *Statistical Rethinking: A Bayesian Course with Examples in R and Stan*.; 2018. doi:10.1201/9781315372495

4. Spiegelhalter DJ, Abrams KR (Keith R., Myles JP. *Bayesian Approaches to Clinical Trials and Health-Care Evaluation*. John Wiley & Sons; 2004.

5. R Core Team. R: A Language and Environment for Statistical Computing. 2020. https://www.r-project.org.

6. Wickham et. al. Welcome to the Tidyverse. *J Open Source Softw*. 2019;4(43):1686. https://doi.org/10.21105/joss.01686.
