# Supplementary material for: Observational study of agreement between attending and trainee physicians on the surprise question: “Would you be surprised if this patient died in the next 12 months?”
Source: PLoS One. 2021 Feb 25;16(2):e0247571. doi: 10.1371/journal.pone.0247571 (PMC7906409; doi:10.1371/journal.pone.0247571)
Supplement: S2 File — (DOCX) [file pone.0247571.s002.docx]

Surprise Project Data Dictionary

2020-12-15

Yarnell et al.

**Demographics**

[1] "X1": Row number

[2] "id” Unique patient ID

[3] "hospital” Hospital identifier

[4] "pair_id" Pair identifier

[5] "sex" Patient sex (M or F)

[6] "age" Patient age in years

[7] "residence" Patient residence prior to hospital admission. OWN = own home, NH = nursing home or longterm care facility, RH = retirement home, NFA = no fixed address, OTH = other such as complex continuing care

[8] "functional" Patient functional status prior to admission. I = independent for ADL and IADL, A = ADL independent but dependent for some IADLs, D = dependent for some ADLs, and U = unknown.

[9] "marital_status" Patient marital status. M = married, D = divorced, S = single, U = unknown, W = widowed / widower

[10] "POA” Patient has a designated power of attorney evident in the chart Y (yes), N (no), or U (unknown)

**Binary diagnosis and comorbidity indicators:** 1 if diagnosis present, 0 otherwise

[11] "Dx_CHF"

[12] "Dx_IHD"

[13] "Dx_COPD"

[14] "Dx_LRTI"

[15] "Dx_cellulitis"

[16] "Dx_UTI"

[17] "Dx_BSI"

[18] "Dx_otherinfection"

[19] "Dx_CVA"

[20] "Dx_seizure"

[21] "Dx_syncope"

[22] "Dx_AKI"

[23] "Dx_lytes"

[24] "Dx_GIB"

[25] "Dx_fall"

[26] "Dx_FTT"

[27] "Dx_cancercomp"

[28] "Dx_delirium"

[29] "Dx_dementia"

[30] "Dx_other"

[31] "pmh_CHF"

[32] "pmh_MI"

[33] "pmh_PVD"

[34] "pmh_HTN"

[35] "pmh_DM"

[36] "pmh_COPD"

[37] "pmh_asthma"

[38] "pmh_dementia"

[39] "pmh_depression"

[40] "pmh_cancer"

[41] "pmh_CKD"

[42] "pmh_arthritis"

[43] "pmh_hepatitis"

[44] "pmh_CVA"

[45] "pmh_other"

[46] "Cr" Patient serum creatinine as of day of data collection (umol/L)

[47] "Alb" Patient serum albumin as of day of data collection (g/L)

**Care preferences:** Coded as Y (yes), N (no), or U (unknown)

[48] "GofC_discussion" Evidence of a goals of care discussion in the chart

[49] "GofC_CPR” Patient is to receive CPR in case of a cardiac arrest.

[50] "GofC_ventilation" Patients is to receive invasive mechanical ventilation if indicated

[51] "GofC_ICU” Patient is for ICU transfer if indicated

[52] "GofC_other” Patient’s preferences include additional information

**Mortality outcomes:** 1 (death), 0 (confirmed alive), NA (unknown)

[53] "death_discharge" hospital discharge mortality

[54] "death_12mo" mortality at 12 months from date of collection

**Surprise Question Responses:** Y (Yes I would be surprised...), N (No I would not be surprised), NA (missing)

[55] "surprise_admission_SMR" Trainee response with respect to hospital mortality

[56] "surprise_12mo_SMR" Trainee response with respect to 12-month mortality

[57] "surprise_admission_AP" Attending physician response with respect to hospital mortality

[58] "surprise_12mo_AP" Attending physician response with respect to 12 month mortality

**Physician length of service:** Duration of service prior to collection date in days

[59] "LOS_SMR" Trainee

[60] "LOS_AP” Attending physician

**Additional binary diagnosis and comorbidity variables:** 1 if present, 0 otherwise

[61] "pmh_afib"

[62] "pmh_vte"

[63] "pmh_hypothyroid"

[64] "pmh_dlp"

[65] "pmh_op"

[66] "pmh_cirrhosis"

[67] "pmh_etoh"

[68] "pmh_gerd"

[69] "pmh_bph"

[70] "pmh_gout"

[71] "pmh_anx"

[72] "dx_sepsis"

**Summary of surprise question responses:** Coded as T (discordance present) or F (discordance absent)

[73] "discordance_admission"

[74] "discordance_12mo"

**Additional length of stay information**

[75] "LOS" Patient length of stay in days prior to data collection
